# Microbial and metabolomic analysis of gingival crevicular fluid in general chronic periodontitis patients: lessons for a predictive, preventive, and personalized medical approach

**DOI:** 10.1007/s13167-020-00202-5

**Published:** 2020-04-16

**Authors:** Jun Pei, Fei Li, Youhua Xie, Jing Liu, Tian Yu, Xiping Feng

**Affiliations:** 1 Department of Preventive Dentistry, Shanghai Ninth People’s Hospital, College of Stomatology, Shanghai Jiao Tong University School of Medicine, Shanghai, 200000 China; 2 National Clinical Research Center for Oral Diseases, Shanghai, 200000 China; 3 Shanghai Key Laboratory of Stomatology & Shanghai Research Institute of Stomatology, Shanghai, 200000 China; 4 Key Lab of Medical Molecular Virology, School of Basic Medical Sciences, Fudan University, Shanghai, 200000 China

**Keywords:** Predictive preventive personalized medicine, Periodontitis, Gingival crevicular fluid, Microbial communities, Metabolites, Multi-omics, Molecular biomarkers, Multi-level diagnostics, Therapeutic targets

## Abstract

**Objectives:**

General chronic periodontitis (GCP) is a bacterial inflammatory disease with complex pathology. Despite extensive studies published on the variation in the oral microbiota and metabolic profiles of GCP patients, information is lacking regarding the correlation between host-bacterial interactions and biochemical metabolism. This study aimed to analyze the oral microbiome, the oral metabolome, and the link between them and to identify potential molecules as useful biomarkers for predictive, preventive, and personalized medicine (PPPM) in GCP.

**Methods:**

In this study, gingival crevicular fluid (GCF) samples were collected from patients with GCP (*n* = 30) and healthy controls (*n* = 28). The abundance of oral microbiota constituents was obtained by Illumina sequencing, and the relative level of metabolites was measured by gas chromatography-mass spectrometry. Full-mouth probing depth, clinical attachment loss, and bleeding on probing were recorded as indices of periodontal disease.

**Results:**

The relative abundances of 7 phyla and 82 genera differed significantly between the GCP and healthy groups. Seventeen differential metabolites involved in different metabolism pathways were selected based on variable influence on projection values (VIP > 1) and *P* values (*P* < 0.05). Through Spearman’s correlation analysis, microorganisms, metabolites in GCF, and clinical data together showed a clear trend, and clinical data regarding periodontitis can be reflected in the shift of the oral microbial community and the change in metabolites in GCF. A combination of citramalic acid and N-carbamylglutamate yielded satisfactory accuracy (AUC = 0.876) for the predictive diagnosis of GCP.

**Conclusions:**

Dysbiosis in the polymicrobial community structure and changes in metabolism could be mechanisms underlying periodontitis. The differential microorganisms and metabolites in GCF between periodontitis patients and healthy individuals are possibly biomarkers, pointing to a potential strategy for the prediction, diagnosis, prognosis, and management of personalized periodontal therapy.

**Electronic supplementary material:**

The online version of this article (10.1007/s13167-020-00202-5) contains supplementary material, which is available to authorized users.

## Introduction

General chronic periodontitis (GCP) is a bacterial inflammatory disease that is induced and maintained by polymicrobial biofilm in subgingival areas; intricate interactions of the microbial communities with the host subvert the host’s homeostasis, disrupting tissue attachment and destroying the supporting structures of the teeth [1, 2]. Periodontitis affects the majority of adults worldwide and may cause various systemic diseases, including diabetes [3] and cardiovascular disease [4]. Therefore, a predictive medicine approach for GCP prevention at the early stage is one of the leading directions of research, and advanced measures for monitoring and analysis are necessary for an in-depth understanding of molecular mechanisms and for the discovery of therapeutic targets for predictive preventive personalized medicine (PPPM) in GCP [5]. Recently, consistent and rigorous effort has been devoted to investigating biomarkers of periodontal pathogenesis through omics technology.

The various microbiota constituents inhabiting the mouth, including at least 400 to 700 prevalent taxa [6, 7], contribute significantly to maintaining the oral and extraoral health of the host [8]. Currently, classification and prediction of host status based on the human microflora has become an important goal of human microbiome projects worldwide [9, 10]. Researchers have increasingly agreed with the hypothesis from an ecological perspective to explain the mechanism of periodontal disease occurrence and development [11], and complicated interactions between oral health and multiple diseases [12, 13]. Under this condition, next-generation sequencing (NGS) technology provides an advanced scientific technique in terms of detecting, identifying, and classifying the oral microbial community [14], and has led to the validation and enhanced understanding of periodontitis prediction conforming to the ecological plaque hypothesis.

Metabonomics has shown interesting capabilities in diagnosing several diseases [15–18] and describing individual metabolic phenotypes in humans [19, 20]. In recent years, several studies have made significant contributions to understanding the biochemical network and pathway in periodontal diseases, pointing out a series of recognized diagnostic biomarkers: enzymes of host or bacterial origin, proteins, inflammatory mediators, collagen and bone degradation products, and DNA of host or bacterial origin [21–26]. Oral metabolomics has attracted much attention in the diagnosis of periodontitis since metabolites are the end products of biological processes by which genomic information is implemented; therefore, they are considered to accurately reflect phenotype differences [27].

At the interface of the epithelia and bacterial plaque is plasma-derived gingival crevicular fluid (GCF). When the tissue is inflamed, this fluid changes to an exudate [2]. Since GCF can be collected noninvasively and is a low-stress, cost-effective, and site-specific collection strategy, it is an ideal tool to detect host-bacterial interactions [28] and to reflect the severity of periodontal inflammation originating from host cells and the numerous microbes harbored in inflamed periodontal pockets. Although there is a wealth of information published on the variation in the oral microbiota and metabolic profiles of GCP, respectively, the relationship between host-bacterial interactions and biochemical metabolism has not been identified. In this study, we conducted a case-control multi-omics analysis of GCF samples from 58 participants (28 healthy controls and 30 patients with GCP) using 16S rRNA and gas chromatograph-mass spectrometry (GC-MS) with multivariate statistical techniques. This study aimed to broaden our understanding of the oral microbiome and metabolism in GCP patients and to provide an option for detailed assessment of pathologic conditions. Integration of microbial data and metabolomic data with useful clinical information will offer more valuable information for PPPM in GCP.

## Materials and methods

### Experimental design and subject selection

This study was approved by the Institutional Ethics Committee of Ninth People’s Hospital, Shanghai Jiao Tong University School of Medicine (issuing number, 201841). All subjects were informed of the purpose of the study and signed an informed consent form at the first visit before enrolment.

A total of 58 subjects who were referred to the Department of Preventive Dentistry, Ninth People’s Hospital Affiliated with Shanghai Jiao Tong University School of Medicine from October to December 2018 were enrolled in this cross-sectional study. Thirty patients with moderate and severe GCP were selected according to the criteria outlined in the World Workshop in Periodontology [29]. GCP was diagnosed with a full-teeth probing examination and panoramic X-ray. All patients had four or more teeth showing at least one site with a probing depth (PD) ≥ 4 mm, a clinical attachment level (CAL) ≥ 3 mm at the same site, and the presence of bleeding on probing (BOP). Twenty-eight healthy controls had a PD ≤ 3 mm and CAL < 1 mm for all teeth. To be included in the study, all subjects were required to fill in a questionnaire and have a minimum of 20 natural teeth (excluding third molars). The exclusion criteria were (a) systemic disease; (b) orthodontic treatment before or periodontal therapy within the past 3 months; (c) use of antibiotics within the past 3 months; (d) pregnant, nursing or taking hormonal contraceptives; and (e) smoking.

### Periodontal examinations and sample collection

Periodontal examination data were recorded on a clinical record for all teeth excluding third molars. PD (measurement from the gingival margin to the total PD) and CAL (measurement from the cemento-enamel junction to the total PD) were assessed at six sites per tooth (mesiobuccal, buccal, distobuccal, distolingual, lingual, and mesiolingual). BOP was evaluated using a dichotomous index (presence or absence of bleeding) and was demonstrated as the percentage of surfaces showing bleeding. The examinations were completed by one trained and calibrated examiner.

GCF samples were collected twice from the patients with GCP at the deepest PD site of every quarter. For the healthy controls, the buccal site of the 4 first molars was the specified collection points. A total of 8 strips were collected from each subject. Before sample collection, the supragingival plaque was gently removed, and the tooth surface was air-dried and isolated using clean cotton rolls. A Periopaper® (Oraflow Inc., NY, USA) filter strip was inserted gently, the strip was held in the gingival sulcus for 30 s, and volume was determined by a precalibrated Periotron 8000® (Oraflow Inc., Plainview, NY, USA). The strips were stored separately in two 1.5 mL Eppendorf (EP) tubes and stored at − 80 °C until further use. One was used for 16S rRNA sequencing, and the other was used for metabolic assessment.

### Sample preparation and 16S rRNA amplicon sequencing

To prepare the internal standard, 10 μL of 2-chloro-l-phenylalanine (0.3 mg/mL) was added into EP tubes with the samples and dissolved in methanol. Afterwards, an ice-cold mixture of methanol and water (methanol:water = 4:1) was added. After 5 min of vortexing, the solution was centrifuged (20,000×*g*, 10 min) at 4 °C. The remaining steps were carried out as described in the literature [30]. Eventually, the solutions were incubated at room temperature for 30 min.

Total bacterial genomic DNA samples were isolated using Fast DNA SPIN extraction kits (MP Biomedicals, Santa Ana, CA, USA) following the manufacturer’s instructions and stored at − 20 °C prior to further analysis. The quantity and quality of extracted DNA were tested using a NanoDrop ND-1000 spectrophotometer (Thermo Fisher Scientific, Waltham, MA, USA) and 1% agarose gel electrophoresis, respectively.

PCR amplification of the bacterial 16S rRNA gene V3–V4 region was conducted using universal primers (338F: 5′ -GTACTCCTACGGGAGGCAGCA-3′, 806R: 5′ -GTGGACTACHVGGGTWTCTAAT-3′). Sample-specific 7-bp barcodes were incorporated into the primers for multiplex sequencing. Thermal cycling consisted of initial denaturation at 98 °C for 2 min; followed by 25 cycles consisting of denaturation at 98 °C for 15 s, annealing at 55 °C for 30 s, and elongation at 72 °C for 30 s; with a final extension at 72 °C for 5 min. PCR products were purified with Agencourt AMPure Beads (Beckman Coulter, Indianapolis, IN, USA) and quantified using a PicoGreen dsDNA Assay Kit (Invitrogen, Carlsbad, CA, USA). After the individual quantification step, amplicons were pooled in equal amounts, and pair-end 2 × 300 bp sequencing was performed at Shanghai Personal Biotechnology Co., Ltd. (Shanghai, China) using the Illumina MiSeq platform with a MiSeq Reagent Kit v3.

### Sample preparation and metabolic analysis of GCF

One piece of the sample paper was placed into a 2 mL EP tube, GCF was extracted with 300 μL of methanol, and 10 μL of adonitol (0.5 mg/mL stock in dH_2_O) was added as an internal standard. Samples were oscillated for 5 min, ultrasound-treated for 10 min (incubated in ice water), and centrifuged for 15 min at 12,000 rpm and 4 °C, and the supernatant (280 μL) was transferred into a fresh 1.5 mL EP tube. Then 300 μL of methanol was added, and the previous steps were repeated. The supernatants were then combined and vortex mixed for 30 s before 500 μL of the supernatant was transferred into a fresh 1.5 mL EP tube; 60 μL from each sample was taken and pooled as a quality control (QC) sample. The remaining sample was dried completely in a vacuum concentrator without heating; 20 μL of methoxyamination hydrochloride (20 mg/mL in pyridine) was added and incubated for 30 min at 80 °C; 30 μL of the BSTFA reagent (1% TMCS, *v*/v) was added to the sample aliquots and incubated for 1.5 h at 70 °C; and 5 μL of FAMEs (in chloroform) was added to the QC sample when cooling to room temperature. All samples were analyzed by a gas chromatograph system coupled with a Pegasus HT time-of-flight mass spectrometer (GC-TOF-MS). The system utilized a DB-5MS capillary column coated with 5% diphenyl cross-linked with 95% dimethylpolysiloxane (30 m × 250 μm inner diameter, 0.25 μm film thickness; J&W Scientific, Folsom, CA, USA).

### Statistical analysis

16S rRNA sequence data analyses were performed using mainly the QIIME and R packages (v3.2.0). Operational taxonomic unit (OTU)–level α diversity indices were calculated using the OTU table in QIIME. α diversity indices, including abundance-based coverage estimator (ACE), the Chao1 richness estimator, Shannon-Wiener diversity index, and Simpson’s index, were calculated to compare the microbial communities based on their diversity and phylogenetic structure. β diversity analysis was performed using UniFrac distance metrics and visualized via nonmetric multidimensional scaling (NMDS). Partial least squares discriminant analysis (PLS-DA) was also introduced as a supervised model to reveal the microbiota variation among groups, using the “plsda” function in the R package “mixOmics.” The significance of differentiation of the microbiota structure among groups was assessed by permutational multivariate analysis of variance (PERMANOVA) and analysis in Adonis. The taxonomy compositions and abundances were visualized using GraPhlAn. Taxon abundances at the phylum, class, order, family, and genus levels were statistically compared among samples or groups by Metastats and visualized as violin plots. Microbial functions were predicted using phylogenetic investigation of communities by reconstruction of unobserved states (PICRUSt, http://huttenhower.sph.harvard.edu/galaxy) with the Kyoto Encyclopedia of Genes and Genomes (KEGG) database.

The GC-MS data were exported by Chroma TOF 4.3X software (LECO, St Joseph, MI, USA); the LECO-Fiehn Rtx5 database was used for raw peak exaction, data baseline filtering and calibration of the baseline, peak alignment, deconvolution analysis, peak identification, and integration of the peak area. Both the mass spectrum match and retention index match were considered in metabolite identification. Peaks detected in < 50% of QC samples or RSD ˃ 30% in QC samples were removed. The resulting data were analyzed by SIMCA-P (version 14.0; Umetrics, Umeå, Sweden). The population distribution among all samples and the stability of the overall analysis process were assessed using principal component analysis (PCA). Orthogonal partial least squares discriminant analysis (OPLS-DA) was used to evaluate the total differences among the groups. Variable influence on projection (VIP) values larger than 1.0 and *P* values (2-tailed Student’s *t* test) less than 0.05 were used to determine differential metabolic profiles. The quality of the PCA and OPLS-DA was evaluated by the values of R2X or R2Y and Q2. Values for the area under the curve (AUC) of the receiver operating characteristic curve (ROC) were used to assess the diagnostic ability of candidate metabolites for diagnosis of moderate or severe periodontitis.

A heat map of Spearman’s rank correlation coefficient was used to illustrate the relationships among microbial communities, metabolites and clinical indices.

### Data availability

The raw sequences of human GCF samples were deposited at the NCBI Sequence Read Archive under SRA Accession no. SRP226726.

## Results

### General demographic and clinical characteristics of the subjects

A total of 58 individuals were enrolled in this study. There was no significant difference in age or sex between the two groups. The PD, the CAL, and the prevalence of BOP of patients were significantly higher in the GCP group than in the control group (*P* < 0.01, Table 1).

**Table 1:** Demographic and clinical characteristics of the subjects

Clinical parameters	Healthy control	Moderate-severe periodontitis
Number of participants	28	30
Age (range)	35.7 (24.0–46)	39 (28–51)
Gender, female	19	17
PPD (mm)^a^	2.5 ± 0.4	3.7 ± 0.5
CAL (mm) ^a^	0.5 ± 0.4	1.9 ± 0.6
BOP (%)^a^	30 ± 19	73 ± 25

^a^*P* < 0.01, Student’s *t* test

### Changes in phylogenetic composition and structure in periodontal microbial communities of GCF

Following 16S rRNA gene sequencing of 116 GCF samples from 58 individuals (60 samples from 30 chronic periodontitis individuals and 56 samples from 28 controls), 2,290,279 high-quality reads were obtained after quality filtration. An ultimate total of 5681 OTUs were found at a 97% identity cut-off among all samples.

**Fig. 1:**
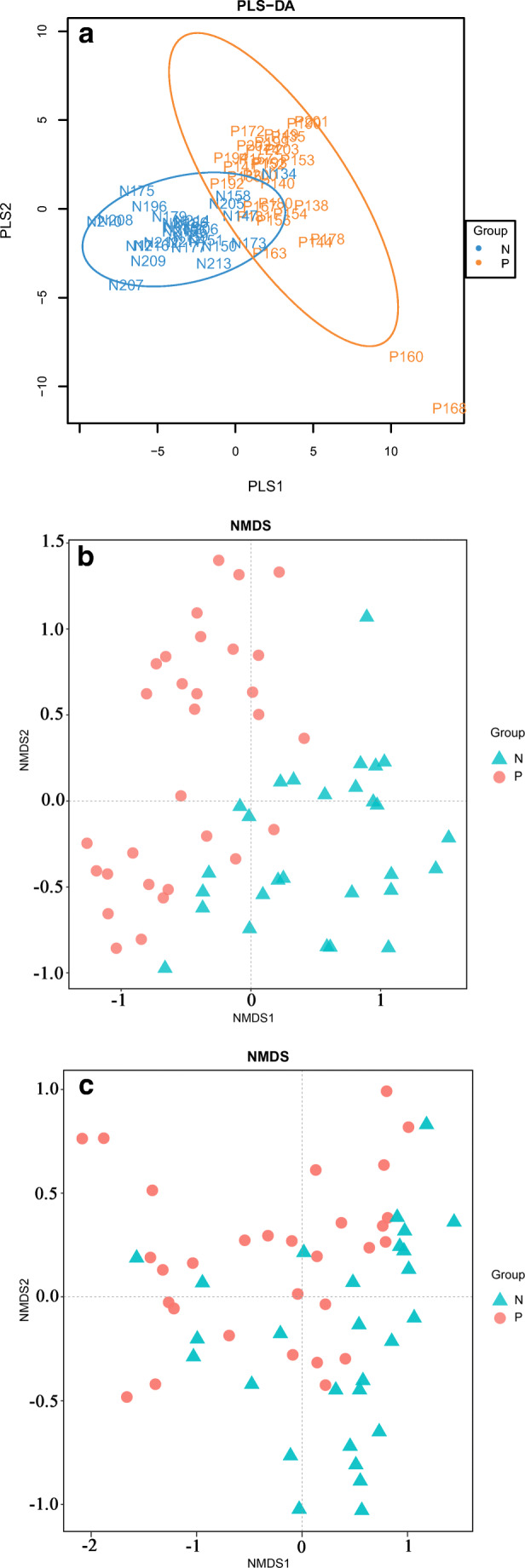
Comparisons of the phylogenetic structure and composition between the microbial communities of patients with GCP (Group P) and healthy controls (Group N). Statistical significance was examined using the Adonis method with 999 permutations. **a** Partial least squares discriminant analysis (PLS-DA) consisted of a supervised model to reveal microbiota variation among groups. The results demonstrated that the sample grouping model was discriminatory. **b** Nonmetric multidimensional scaling (NMDS) based on unweighted UniFrac distances for bacterial communities between the two groups, *P* = 0.001. **c** NMDS based on weighted UniFrac distances between the two groups, *P* = 0.002

According to the given sample distribution information and species abundance matrix, the community structure data were discriminated and analyzed by PLS-DA. If samples belonging to the same group are closer to each other and the points belonging to different groups are farther from each other, then the classification model is better. The results demonstrated that the sample grouping model was effective (Fig. 1a).

To characterize dysbiosis in the oral microbial communities of periodontal disease patients compared to those of healthy individuals, we analyzed α and β diversities of the microbiota to evaluate their overall compositional richness and structural features. To illustrate the microbial community richness, evenness, and species diversity, α diversity indices, including the Chao1 index, ACE index, Shannon index, and Simpson index, were used, with no significant differences between the two groups (Supplementary Fig. S1, *P* > 0.05). However, β diversity analysis according to NMDS and based on unweighted and weighted UniFrac distances at the OTU level demonstrated a statistically significant separation of the two groups (Adonis, *P* < 0.01; Fig. 1b, c), suggesting different overall microbial community structures. The closer the distance between samples shown in the picture, the more similar the microbial community structures are. The results demonstrated that the GCF microbial community structure changed significantly between healthy controls and periodontitis patients.

**Fig. 2:**
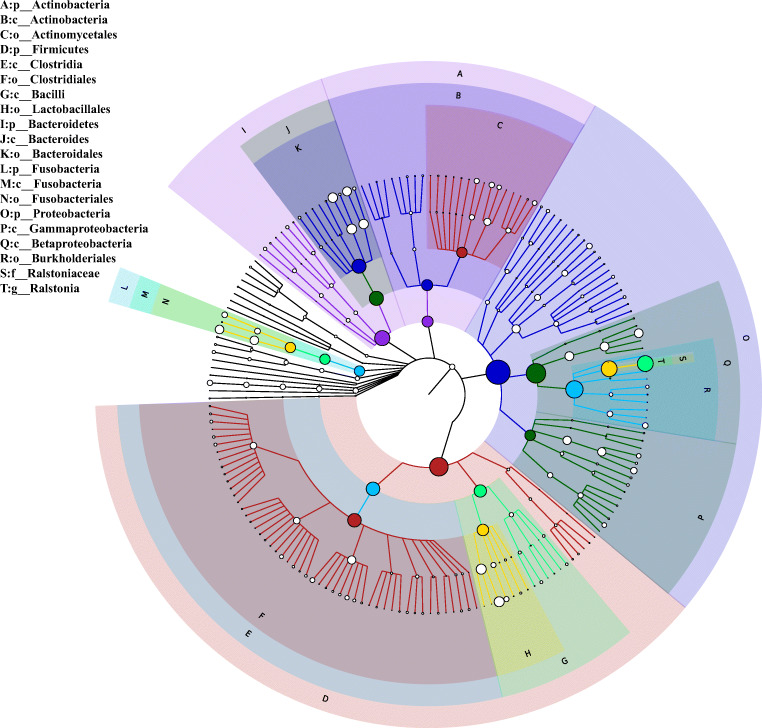
Visualization of taxa on a phylogenetic tree from phylum to genus levels (arranged from the inner circle to the outer circle), as analyzed using GraPhlAn. The node size reflects the mean relative abundance of the taxon. The top 20 dominant taxa are identified in the legend

By analyzing of all the GCF samples, a total of 13 phyla, 23 classes, 40 orders, 85 families, and 177 genera were detected. From the overall GCF samples, the dominant phyla included Proteobacteria, Firmicutes, Bacteroidetes, Actinobacteria, Fusobacteria, Spirochaetes, and Synergistetes (> 99% of the overall abundance). *Ralstonia*, *Streptococcus*, *Porphyromonas*, *Prevotella*, *Neisseria*, *Fusobacterium*, *Haemophilus*, *Sphingomonas*, *Leptothrix*, *Leptotrichia*, *Kocuria*, *Treponema*, *Corynebacterium*, *Lactobacillus*, *Veillonella*, *Escherichia*, *Actinomyces*, *Aggregatibacter*, *Rothia*, *Selenomonas*, *Lachnospiraceae*[*G-2*], *and Bacteroidetes*[*G-5*] were the top 22 most abundant genera, which composed 74.5% of the overall abundance (Supplementary Fig. S2a). A phylogenetic tree constructed with GraPhlAn at various classification levels is shown in Fig. 2, which can quickly identify dominant microbial taxa from phylum to genus. When the two group samples were analyzed separately, the precise proportion of dominant taxa was somewhat different between periodontitis samples and healthy samples (Supplementary Fig. S2b).

**Fig. 3:**
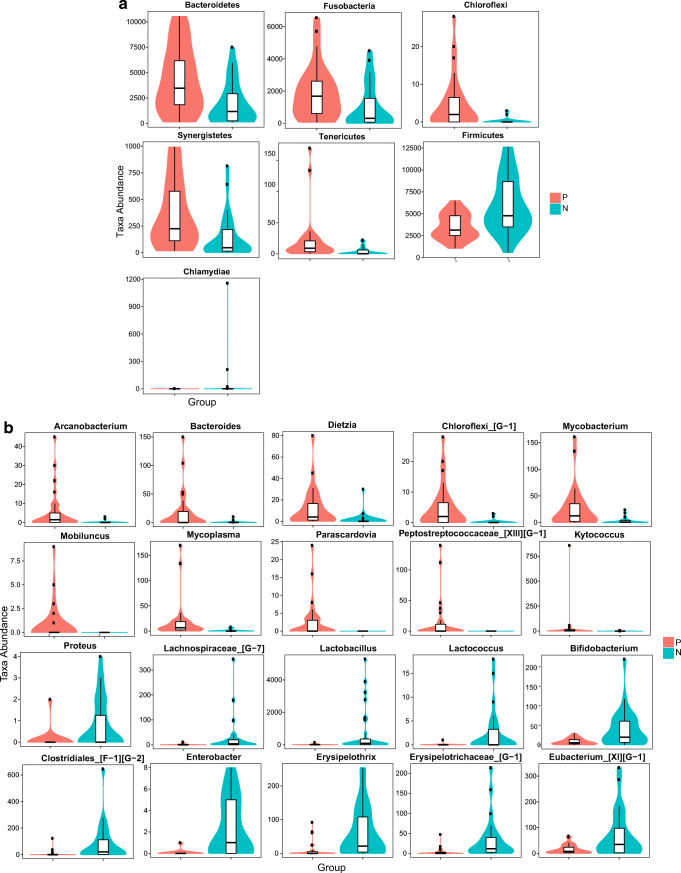
Taxon abundances at the phylum (**a**) and genus (**b**) levels were statistically compared between patients with GCP (Group P) and healthy controls (Group N) by Metastats. The violin plots show all phyla and the top 20 genera with significant differences

Metastats analysis was performed to compare the differences in taxa (absolute abundance) between the two groups at the phylum and genus levels. A total of 7 phyla and 82 genera revealed different abundances (Supplementary Table S1). Samples are displayed in the form of violin diagrams combined with box-line diagrams, and violin diagrams can visually display the distribution characteristics of data. At the phylum level, the relative abundances of Chloroflexi, Synergistetes, Tenericutes, Bacteroidetes, and Fusobacteria in GCP patients were significantly higher than those in the healthy controls. However, a relatively higher abundance of Firmicutes and Chlamydiae was observed in healthy controls compared with that in periodontal patients (Fig. 3a). At the genus level, the top 20 taxa with the most significant differences between the two groups are listed in Fig. 3b. *Arcanobacterium*, *Bacteroides*, *Dietzia*, *Chloroflexi_*[*G-1*], *Mycobacterium*, *Mobiluncus*, *Mycoplasma*, *Parascardovia*, *Peptostreptococcaceae_*[*XIII*][*G-1*], and *Kytococcus* were significantly enriched in the periodontal disease patients compared with those in the healthy controls. In contrast, several genera, namely, *Proteus, Lachnospiraceae_*[*G-7*], *Lactobacillus*, *Lactococcus*, *Bifidobacterium*, *Clostridiales*_[*F-1*][*G-2*], *Enterobacter*, *Erysipelothrix*, *Erysipelotrichaceae*_[*G-1*], and *Eubacterium*_[*XI*][*G-1*], exhibited lower proportions in periodontal disease patients than in the controls. Together, these data reveal microbial changes in the GCF microbiome in periodontal disease individuals, suggesting a state of microbial dysbiosis.

### Functional variation in the periodontal microbiota

Another focus of this study was to disclose the functional variation in the periodontal GCF microbial community. Therefore, the microbiota-derived pathways were predicted by the PICRUSt algorithm with the KEGG database, and functional abundance was compared between the periodontal disease patients and healthy control groups. In total, 41 detailed pathways were characterized in the present study. For the study of microbial ecology, the metabolic function of the microflora is the most important. According to the predicted abundance distribution of each functional group in each sample, a violin diagram was drawn (Supplementary Fig. S3). Specifically, the functional changes in periodontal samples included an increase in basic metabolism (e.g., energy, cofactor, and vitamin metabolism), enzyme families, glycan biosynthesis and metabolism, and biosynthesis of secondary metabolites. In contrast, a loss of carbohydrate metabolism was observed in periodontal communities. In conclusion, the results not only demonstrated compositional dysbiosis but also predicted metabolic functional disturbance.

### Shifts in metabolomic profiles of GCF samples

To investigate the extent to which the altered microbiome in the periodontal disease patients was associated with metabolites, we performed nontargeted metabolomics profiling of 174 GCF samples from cases and controls.

**Fig. 4:**
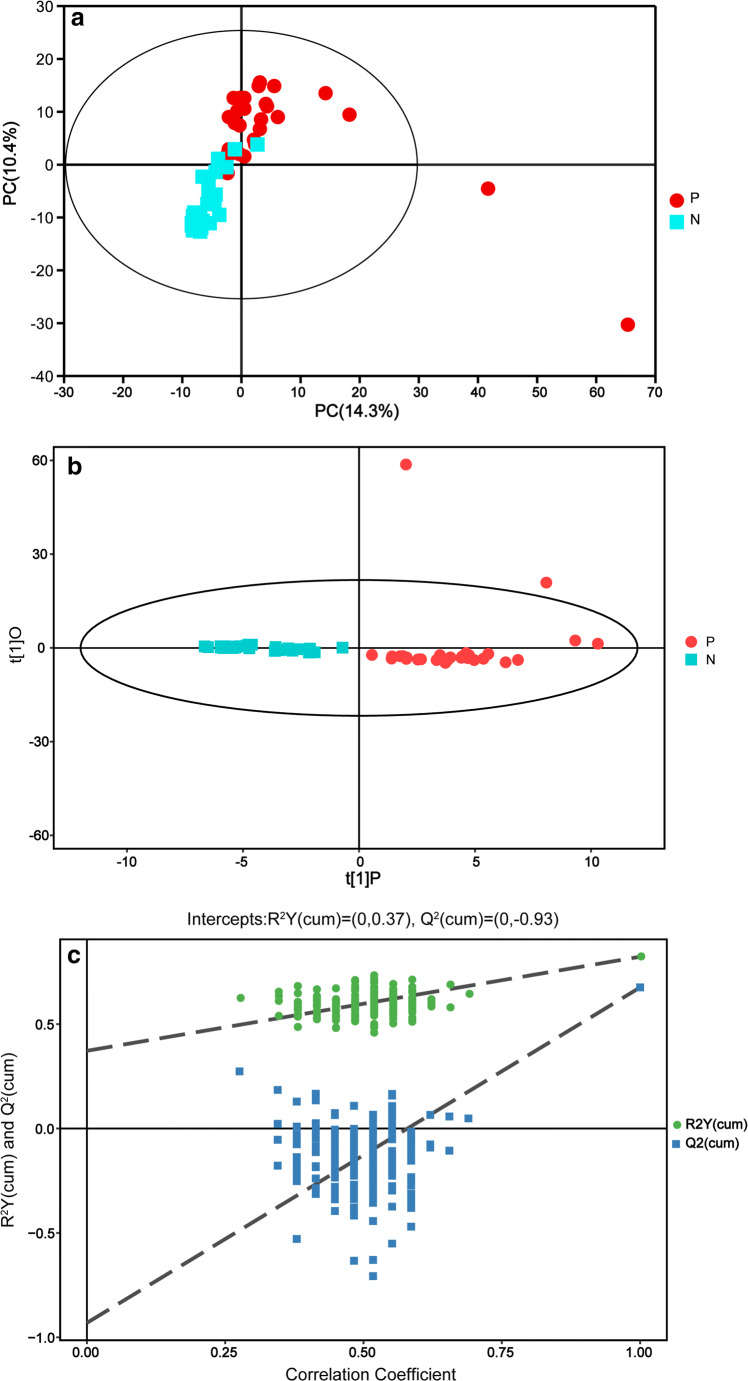
Typical gas chromatography-mass spectrometry scores plots. **a** Principal component analysis (PCA) plot model of gingival crevicular fluid (R2X = 0.508). **b** The orthogonal least square-discriminative analysis (OPLS-DA) model for the GCP group (P) and healthy group (N) (R2Y = 0.823, Q2 = 0.676). **c** OPLS-DA 200 permutation testing: (R2Y = 0. 37, Q2 = − 0.93). The generated explained variation values and the predictive capability indicate the excellence in modeling and prediction, with clear discrimination between the GCP and healthy groups

After removing the internal standards and pseudopositive peaks and combining the peaks from the same metabolite, a total of 147 qualitative metabolites were obtained. Both the plot of PCA scores (Fig. 4a) and the OPLS-DA model (Fig. 4b, c) demonstrated satisfactory modeling and predictive abilities. Compared with controls, periodontal disease individuals displayed pronounced metabolic alterations. Among them, 17 metabolites were selected based on standards among the differential variables with VIP values > 1 in the OPLS-DA and *P* values in Student’s *t* test < 0.05 (Table 2). The GCF metabolites that differed most significantly in periodontal disease individuals relative to those in healthy controls included elevated glycine-d5 (fold change (FC) = 20.38), N-carbamylglutamate 2 (FC = 9.83), and fructose 1 (FC = 5.92) and depleted lactamide 2 (FC = 0.65), O-phosphoserine 1 (FC = 0.71), and 1-monopalmitin (FC = 0.72).

**Table 2:** Differential metabolites between periodontitis and healthy controls

Peak	Mean P	Mean N	VIP	*P* value	*Q* value	Fold change	Log_foldchange
Glycine-d5	0.051333284	0.002518903	2.686654583	1.49E-07	3.65352E-06	20.37922203	4.349027073
N-Carbamylglutamate 2	0.001037846	0.000105564	2.630242979	4.21401E-09	2.5869E-07	9.831450847	3.297404334
Fructose 1	0.002357247	0.000398217	2.398089278	0.001428095	0.017702445	5.919500674	2.565475486
2-Butyne-1,4-diol	0.006857957	0.001194529	2.879871275	7.8385E-09	3.84952E-07	5.741140703	2.521337413
5-Dihydrocortisol 3	0.000558885	9.8688E-05	1.227185617	0.002842353	0.027874389	5.663150523	2.501604876
N-Acetyl-beta-D-mannosamine 1	0.000319463	6.02506E-05	2.178661077	0.001755285	0.020494345	5.302237027	2.406601165
4-Hydroxyphenylacetic acid	0.002672063	0.000709054	1.96772351	0.003112315	0.029393691	3.768488233	1.913985888
Citramalic acid	0.002650632	0.000758167	2.984629212	9.68575E-08	2.91543E-06	3.49610666	1.8057492
Uracil	0.007927636	0.003428043	1.617699878	0.000549653	0.008165377	2.312583439	1.20950542
beta-Glutamic acid 1	0.002995735	0.002020754	1.293178024	0.020879645	0.099659996	1.48248402	0.568016553
Monoolein	0.007769257	0.005862759	1.627516713	0.02848719	0.117778288	1.325187884	0.406196919
Methylmalonic acid	0.029476006	0.033940995	1.664596792	0.018708654	0.094957487	0.868448476	−0.203487837
Thymidine 3	0.001377672	0.001740672	1.096219131	0.009559978	0.066454136	0.791459902	−0.337411835
Octadecanol	0.001733205	0.002235684	1.739203072	0.03129264	0.123639982	0.775245838	−0.367274219
1-Monopalmitin	0.000614955	0.000854443	1.157584723	0.008945999	0.063789651	0.719715264	−0.474501838
O-Phosphoserine 1	0.00071198	0.001001571	1.001597189	0.010134474	0.068828752	0.710863854	−0.492354816
Lactamide 2	0.000382039	0.000585076	1.755482094	0.009031916	0.064170744	0.652973669	−0.614903278

**Fig. 5:**
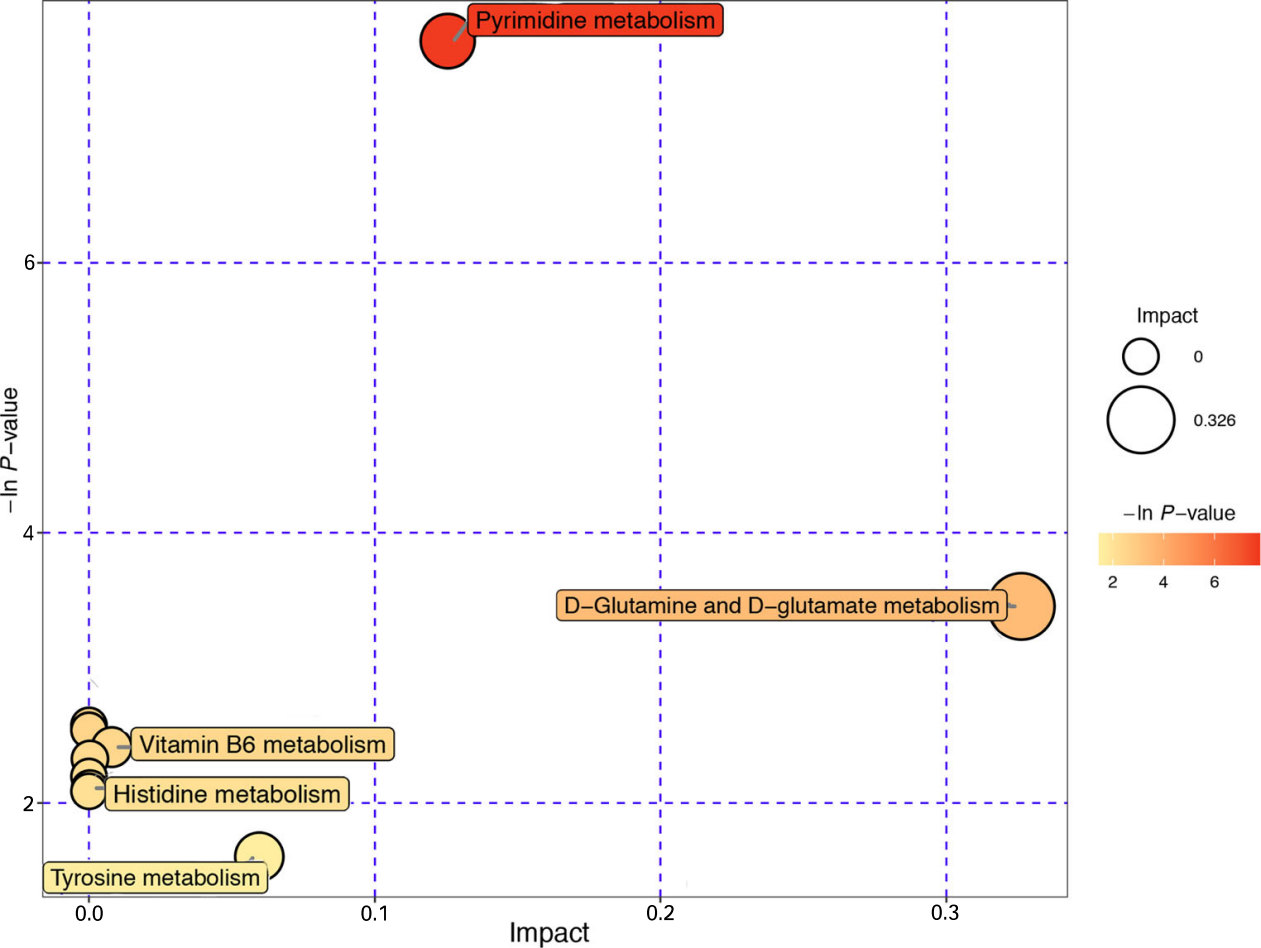
Bubble plot of metabolite set enrichment analysis (MSEA) and topology analysis of differential metabolites. The abscissa and bubble size indicate the influencing factor of the pathway in the topology analysis (the larger the size is, the larger is the influencing factor); the *Y*-axis and bubble color indicate the *P* value in enrichment analysis (the darker the color is, the smaller is the *P* value)

Complex metabolic reactions and their regulation in organisms are not carried out independently. Their interaction and mutual regulation eventually lead to systematic changes in metabolites. To further characterize the signatures of the metabolites, we conducted metabolite set enrichment analysis (MSEA) and topology analysis, which was able to identify which pathways are overrepresented among differential metabolites in reference to a metabolite set list created on the basis of the KEGG database (Supplementary Table S2). As shown in Fig. 5, pyrimidine metabolism and d-glutamine and d-glutamate metabolism were identified as significantly overrepresented pathways in the periodontal disease group (*P* = 0.000488027 and 0.031594, respectively), which might reflect the metabolic signatures of disease-associated communities. Vitamin B_6_ metabolism, propanoate metabolism, histidine metabolism, and tyrosine metabolism were also related pathways with no significant difference. Differential metabolites, namely, uracil, thymidine, and methylmalonic acid, were involved in pyrimidine metabolism. Glutamic acid was involved in d-glutamine and d-glutamate metabolism.

### Associations among the microbiota, metabolites, and periodontal clinical indices

Through Spearman’s correlation analysis, the correlations between clinical data for periodontitis, the microbiota, and metabolites were reviewed.

**Fig. 6:**
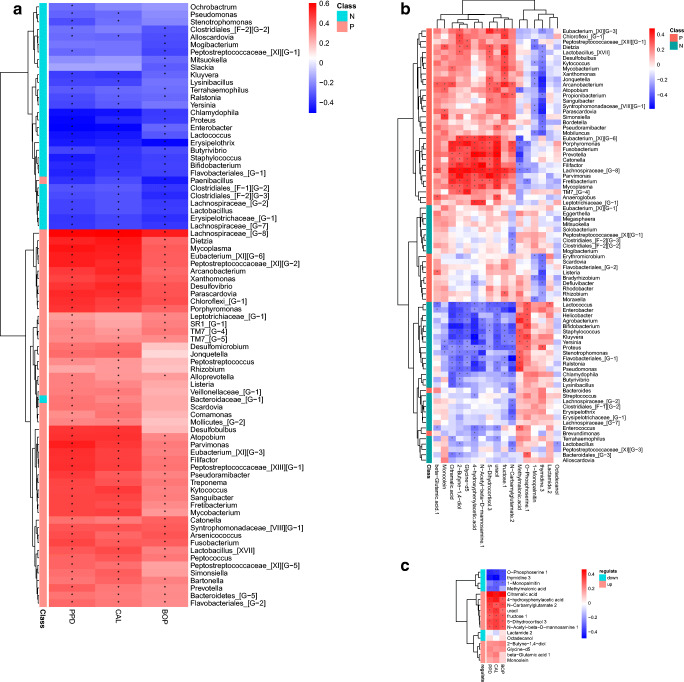
Associations among the microbiota, metabolites, and periodontal clinical indices. **a** Heat map of microbial genera with clinical indices. Spearman’s rank correlation between 80 genera and 3 clinical indices (only genera correlating with at least one clinical index with *P* < 0.05 are shown). **b** Heat map of microbial genera with differential metabolites. Spearman’s rank correlation between 81 genera and 17 differential metabolites. Genera in green and red classes denote control enrichment and periodontitis enrichment, respectively. **c** Heat map of clinical indices with differential metabolites. Spearman’s rank correlation coefficient between 3 clinical indices and 17 differential metabolites. Metabolites in green and red classes denote downregulated and upregulated in the periodontitis group, respectively. *P < 0.05

After analysis, the genera with significant correlations with clinical data are shown in a heat map as ordinates (Fig. 6a). As shown in the figure, there was a strong statistically significant correlation between the bacterial genera detected in the oral cavity and the clinical data of periodontitis, including BOP, CAL, and PD. This result indicated a positive relationship between the periodontal disease patient–enriched genera and the clinical detection data and a negative correlation between the healthy control–enriched genera and the clinical data.

To further explore whether the altered abundance of metabolites correlated with the altered GCF microbiota, covariation between the 82 genera and 17 metabolites that differed between cases and controls was investigated by Spearman’s correlation. Notably, most metabolite levels that were elevated in periodontal disease individuals, including citramalic acid, 2-butyune-1,4-diol, glycine-d5, 4-hydroxyphenylacetic acid, N-acetyl-β-D-mannosamine 1, 5-dihydrocortisol 3, uracil, fructose 1 and N-carbamylglutamate 2, were positively correlated with the majority of periodontal disease–enriched genera and negatively correlated with the majority of healthy control–enriched genera. In contrast, metabolite levels that were depleted in periodontal disease individuals, consisting of methylmalonic acid and O-phosphoserine 1, exhibited positive correlations with many healthy control–enriched genera and negative correlations with periodontal disease–enriched genera. Thymidine 3 and 1-monopalmitin, metabolites whose levels were also depleted in periodontal disease individuals, had a negative correlation and significant difference with most periodontal disease–enriched genera. Specifically, the genera of putative periodontopathic bacteria, such as *Porphyromonas*, *Prevotella*, *Fusobacterium*, and *Filifactor*, demonstrated a close relationship with differential metabolites (Fig. 6b). Taken together, these results suggest that the altered oral microbiota are related to subgingival metabolism to some extent and that the levels of GCF metabolites may reflect changes in the abundance of these bacterial species.

Regarding metabolites, uracil, N-carbamylglutamate 2, N-acetyl-β-D-mannosamine 1, fructose 1, citramalic acid, 5-dihydrocortisol 3, and 4-hydroxyphenylacetic acid were found to be significantly positively linked to increased BOP, CAL, and PD, while the opposite trends were observed for thymidine 3 and O-phosphoserine 1. Furthermore, methylmalonic acid and 1-monopalmitin were negatively correlated with CAL, PD, and BOP, with CAL being significantly negatively correlated (Fig. 6c). Therefore, it is obvious that increased metabolite levels in periodontitis cases were positively correlated with clinical data of periodontitis, while decreased metabolite levels were negatively correlated with clinical data. That is to say, the more the level of increased or decreased metabolites changes, the greater the clinical data and the more obvious the clinical features of periodontitis will be. This result enhanced the change in metabolites in response to the clinical condition of periodontitis.

**Fig. 7:**
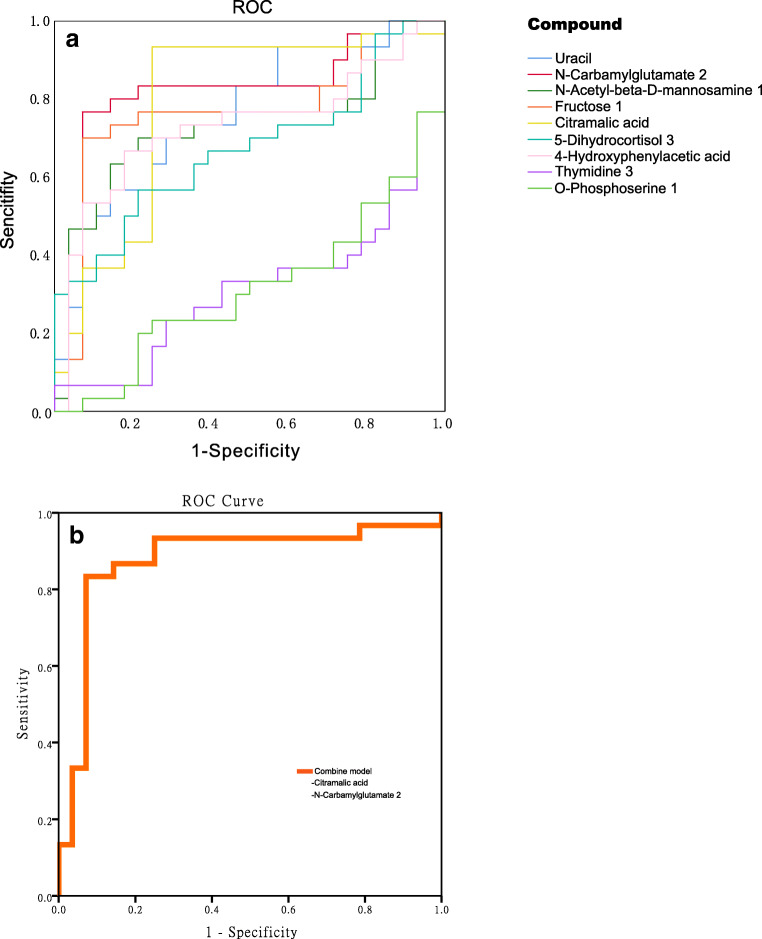
**a** Receiver operating characteristic (ROC) curve of 9 differential metabolites for distinguishing the general chronic periodontitis group from the healthy group. **b** Citramalic acid and N-carbamylglutamate 2 were selected and validated as putative biomarkers, with an area under the curve (AUC) 0.876

The strong positive contribution of the abovementioned 9 metabolites to the prediction of BOP, CAL, and PD suggests their possible application as indicators of periodontal inflammation severity. ROC curves also indicated that the combination of citramalic acid and N-carbamylglutamate 2 yielded satisfactory accuracy for the diagnosis of moderate or severe periodontitis (AUC = 0.876; Fig. 7a, b, Table 3), which may indicate that citramalic acid and N-carbamylglutamate 2 are effective biomarkers in GCF for periodontitis. In sum, these results provide insight into metabolic signatures of periodontal dysbiotic communities and identify potential biomarkers of inflammatory status.

**Table 3:** Area under the ROC

Test result variable	Area	Std. error ^a^	Asymptotic sig. ^b^	Asymptotic 95% confidence interval	
				Lower bound	Upper bound
Uracil	0.76	0.063	0.001	0.636	0.883
N-Carbamylglutamate-2	0.815	0.062	0	0.694	0.937
N-Acetyl-beta-D-mannosamine 1	0.726	0.07	0.003	0.588	0.864
Fructose1	0.768	0.067	0	0.636	0.899
Citramalic acid	0.788	0.065	0	0.661	0.915
5-Dihydrocortisol 3	0.674	0.071	0.023	0.534	0.813
4-Hydroxyphenylaceticacid	0.73	0.069	0.003	0.594	0.866
Thymidine3	0.313	0.071	0.015	0.174	0.452
O-Phosphoserine1	0.315	0.07	0.016	0.178	0.453
Combine model	0.876	0.053	0	0.773	0.98

^a^Under the nonparametric assumption

^b^Null hypothesis: true area = 0.5

## Discussion

### Use of 16S rRNA gene sequencing and GC-MS technologies in periodontitis

Periodontitis is an infectious disease with a complex etiology and is especially relevant to the objectives of PPPM due to its huge impact on people’s social lives [31]. For many years, the study of the bacterial etiology of periodontitis has continued, as a variety of microorganisms have been seen as the most important factor involved in the disease. To promote a reliable method for efficient clinical management, exploring the complex etiology of periodontal disease is imperative [32]. A comprehensive understanding of the ecology of the subgingival microbiota and the interplay between the microbiome composition and metabolomic condition could enable the development of novel prevention and treatment strategies for periodontitis.

Using 16S rRNA gene sequencing and GC-MS technologies, we showed significant phylogenetic differences in subgingival microbial communities and metabolite signatures of host GCF between periodontitis patients and healthy individuals. In general, the 16S rRNA analysis illustrated that the phylogenetic composition and structure of the oral microbiome were distinctly different between periodontal disease and healthy individuals. According to the metabolomic profile analysis, the discrimination obtained from GCF samples demonstrates the existence of a metabonomic signature of GCP disease in GCF. Moreover, the association of the altered microbiota, metabolites, and clinical indices was consistent between the two groups, which indicated that bacteria and metabolism in the subgingival environment were closely related and that this correlation and difference between periodontitis and the health of the microecology were also shown in clinical data. Therefore, the result provided a possible approach to predict and distinguish periodontitis, as metabolites were an indicator of inflammatory status.

### Achievements in the current study: results interpretation of microbial communities

In the current study, α diversity did not identify clear differences between healthy individual samples and periodontitis individual samples. One possible explanation is that the common phyla in the GCF are relatively constant because the major phyla were similar in previous studies. In addition, all individuals in this study came from the same living area, which led to nonsignificant differences in bacterial α diversity between the two groups due to the high similarity of diet and living habits. The β diversity comparisons exhibited significant differences in the microbial community between the periodontitis and healthy groups. As the health status of periodontal tissue changes, the composition of the subgingival plaque community shifted in periodontitis.

At the phylum level, the microbiota from periodontitis had higher proportions of Bacteroidetes, Actinobacteria, Fusobacteria, Spirochetes, and Synergistetes, while the proportions of Proteobacteria and Firmicutes were higher in the microbiota from healthy individuals, which showed that most taxa concurred with previous studies but not at the exact same proportion [33–35] (Supplementary Fig. S2b). *Porphyromonas*, *Prevotella*, *Neisseria*, *Fusobacterium*, *Treponema*, etc., some included well-known destructive periodontal pathogens (*Porphyromonas gingivalis*, *Treponema denticola*, and *Prevotella intermedia*), presented at a higher abundance at the genus level in periodontitis individuals, while *Ralstonia*, *Streptococcus*, and *Haemophilus* showed higher proportions in healthy individuals. Moreover, a Metastats analysis indicated that *Porphyromonas*, *Prevotella*, *Filifactor*, *Fusobacterium*, etc. were significantly enriched in periodontal disease patients (Supplementary Table S1). Our result was in agreement with reported studies. The taxonomic enrichment in these taxa may contribute to the differences in the diversity of GCF samples and, to a certain extent, to the function of the subgingival microbial community between periodontitis and healthy subjects. Some new microorganisms prevalent and strongly associated with disease need further study because they may play a significant role in the development of periodontitis. For instance, researchers proved that *Filifactor alocis* has common characteristics with established periodontal pathogens and potential to cause periodontal tissue destruction [36, 37].

For the study of microbial ecology, we are most concerned about the metabolic function of the microflora. With the development of data analysis technology, we can now predict the metabolic function of bacteria based on the results of 16S rRNA gene sequencing to match the “identity” of species to their “function.” According to this prediction, we can obtain a glimpse of the outline of microbial function and the possible effect of the biochemical activities of bacteria on the host. In our study, metabolic activities, including basic metabolism (e.g., energy, cofactor, and vitamin metabolism), enzyme families, glycan biosynthesis and metabolism, and biosynthesis of secondary metabolites, were upregulated in the microbiota of periodontal disease individuals compared to those in the microbiota of healthy controls. Nevertheless, a decrease in carbohydrate metabolism was observed in periodontal communities. Previous studies found that functional carbohydrate metabolism genes were relatively similar between periodontitis and healthy individuals [38, 39]. Based on our results, it is hypothesized that the community of the periodontal group might utilize sugars absorbed directly instead of biosynthesizing them, so they show a relatively decreased carbohydrate metabolism. In conclusion, the results not only demonstrated compositional dysbiosis but also predicted metabolic functional disturbance.

### Achievements in the current study: results interpretation of metabolites

We successfully screened 17 differential metabolites in GCF samples by GC-MS possibly separating patients with GCP from healthy controls. Many metabolic changes implicated an association with periodontal disease progression.

The synthesis, degradation, and interconversion of DNA, RNA, lipids, and carbohydrates all require the involvement of pyrimidine metabolism. The nucleic acid of periodontal cells can be released when cells are impaired directly by periodontal microbiota or indirectly by the host immune system. Nucleosides and nucleobases are an important nutrient source for bacteria and can be used for nucleic acid biosynthesis or decomposition into carbon and energy sources. As the result of MSEA and topology analysis, pyrimidine metabolism was found to be the most significant pathway involved in GCP (Fig. 5). The elevated level of uracil and the decreased levels of thymidine and methylmalonic acid indicated that pyrimidine metabolism in GCP patients was different than that in healthy controls. Furthermore, uracil had a positive correlation with the periodontal disease–enriched genera and a negative correlation with the healthy control–enriched genera. This relationship between thymidine, methylmalonic acid, and genera was the opposite. It is known that when pathogens invade host cells, they can affect pyrimidine metabolism in the host to create advantageous conditions for proliferation [40]. This finding suggests that the pathogens associated with GCP may alter the metabolism of infected hosts by an as yet unknown mechanism.

Periodontal tissues are rich in proteins. As shown in MSEA and topology analysis, d-glutamine and d-glutamate metabolism, histidine metabolism, and tyrosine metabolism were related pathways involved in the GCF of periodontitis individuals, which affirms findings of previous work that reported increased degradation of macromolecules, including proteins, in individuals with periodontitis [41, 42]. Periodontal microorganisms and host-derived inflammatory proteases can degrade host periodontal proteins into peptides and amino acids that would serve as an abundant energy pool and nutritional resource for microbes [43, 44], ultimately affecting microorganisms and functional structure. Li et al. [39] indicated that subgingival microbiomes might directly absorb some ammonia for physiological activities instead of biosynthesizing it because of oral microbiome genes involved in amino acid synthesis showed a reduced relative abundance in periodontitis. Since periodontal inflammation gives rise to destruction of the connective tissues, it is likely that the elevated level of glycine-d5 and β-glutamic acid in GCF was produced by tissue breakdown.

Reactive oxygen species participate in periodontal destruction during inflammatory periodontal diseases. The imbalance between oxidant and antioxidant activity can be a key factor in the destructive effect of reactive oxygen species [45]. Considering the results, an increased antioxidant activity was observed in the GCF of periodontal individuals, as reflected by the increased concentration of N-carbamylglutamate. Previous evidence has indicated that N-carbamylglutamate can alleviate oxidative stress by enhancing the activities of antioxidant enzymes [46] and improving the nonenzymatic antioxidant content [47] in mammals. Additionally, N-carbamylglutamate can alleviate the inflammatory response by downregulating IL-1b and TNF-a mRNA expression [46]. However, our result was inconsistent with previous studies, which confirmed that periodontal inflammation is closely associated with oxidative stress [48, 49]. An explanation is that in patients suffering different degrees of periodontitis, only those with advanced periodontitis have reduced total antioxidant activity and increased levels of reactive oxygen species [50, 51]. Our samples were taken from patients with moderate to severe periodontitis. Based on clinical data, the average PD and CAL did not indicate severe periodontitis indices.

The metabolism of GCF is closely related to bacterial biochemistry. It is well known that most periodontitis-related bacteria use sugar as an energy and carbon source. The result showing that the level of fructose-1 was higher in patients with GCP than in healthy controls was consistent with a previous metabolomic study of oral biofilms showing elevated fructose-6-phosphate content [52].

As reported, 4-hydroxyphenylacetic acid is produced by *Porphyromonas gingivalis*, an indigenous bacterium in the human oral cavity, as a metabolic end product during the metabolism of phenylalanine and tyrosine [53]. In saliva from healthy individuals, the concentration of 4-hydroxyphenylacetic acid was below 10 μM [54], whereas a 4-hydroxyphenylacetic acid concentration higher than 20 μM was found in periodontitis individuals [55]. The increased population of *Porphyromonas gingivalis* in periodontitis patients may contribute to this increase in 4-hydroxyphenylacetic acid.

### Microbiota and metabolites as tools for predicting periodontal inflammatory status

From the results, there were distinct differences in clinical data, subgingival microorganisms, and metabolites of GCF between the periodontitis group and the healthy control group. Furthermore, correlation heat map analysis of microorganisms, metabolites, and clinical data suggested a concerted trend among them (Fig. 6). Specifically, severe periodontal clinical data were positively correlated with the periodontitis-enriched genera and periodontitis-upregulated metabolites but were negatively correlated with the healthy-enriched genera and periodontitis-downregulated metabolites. Additionally, most periodontitis-upregulated metabolites showed a significantly positive relationship with some periodontitis-enriched genera and a significantly negative relationship with healthy-enriched genera. Similarly, periodontitis-downregulated metabolites had an opposite relationship with the genera. These results confirm that host and oral microorganisms are closely related and interact in the development of periodontitis. Given that the transition from periodontal health to disease is linked to overall imbalance, including altered metabolic signatures in the host and in the periodontal microbial community at the same time, these findings raise the possibility to help survey disease activity and modulate complicated microbial interactions in the formation of a periodontopathic community. In addition, clinical data of periodontitis can be reflected in the shift of the oral microbial community and the change in metabolites in GCF. This result suggested that the microbiota and metabolites might be tools for predicting periodontal inflammatory status. In the current study, a ROC curve was also used to evaluate how these differentially abundant metabolites indicate or further predict periodontitis. A combination of citramalic acid and N-carbamylglutamate yielded satisfactory accuracy (AUC = 0.876) for predictive diagnosis of periodontitis in our study, whereas another study found totally different metabolites for diagnosis [56]. This inconsistency among different studies may be caused by different study samples and the inherent biological diversity between individuals. These indicators require further investigation to prove clinical significance.

### Association of oral microbiota of periodontitis and global health

Numerous reports have demonstrated the involvement of oral microbiota in the pathogenesis of systemic diseases, such as cardiovascular diseases [57], rheumatoid arthritis [58], and intestinal inflammation [59]. In an immunocompromised population, the entry of oral opportunistic or pathogenic bacteria into the blood circulation through the oral mucosal barrier could lead to abnormal local or systemic immune and metabolic responses and nutrient digestion [60–62]. Conversely, systemic multifactorial diseases could result in direct modification of the oral microbiota.

On the side of immunity, the immune defense system, including innate lymphoid cells and pattern recognition receptors, as well as tolerance, is highly complex, and it is important to preserve the proper microbiological and functional homeostasis of the oral cavity. Inappropriate host response or insufficient immune response will either cause tissue damage or permit microbial overgrowth and invasion. When oral pathogenic bacteria overgrow due to poor oral hygiene, the responses of both innate and adaptive immunity increase, which can also give rise to more extensive collateral tissue damage. Periodontal diseases could appear as a result [2]. In turn, oral diseases are linked to many immune-related diseases (e.g., systemic sclerosis, Sjögren’s syndrome, and rheumatoid arthritis) [63, 64] via the microbiome of the oral cavity.

Systemic metabolic health is also closely related to periodontal disease. Dental and oral bacteria are frequently early indicators and risk factors for obesity [65]. Goodson et al. [66] suggested that the oral bacteria may increase metabolic efficiency and energy metabolism by facilitating insulin resistance, affecting weight increase and the development of obesity. Rostyslav et al. [67] found that (1) monosodium glutamate–induced obesity triggers periodontal tissue alterations; and (2) nanoceria contributes to the corrections of pathological changes in periodontal tissues via balancing protein-inhibitory capacity and reducing the depolymerization of fucosylated proteins and proteoglycans and antioxidative activity in glutamate-induced obese rats.

The mouth, as the sole natural entrance for food, controls the intake and initial interaction of what could become the gut microbiota. Accordingly, the oral microbiome may have an altered structure, composition, and function because of gut microbiota changes due to host-microbiome interactions. Some studies have described variation in the oral microbial diversity and composition in human patients with inflammatory bowel diseases [68–70]. Xun et al. [71] explored stratification of patients and biomarkers indicative of inflammatory bowel diseases depending on oral microbial profiles. Furthermore, it is well known that probiotics and prebiotics can manipulate the microbiota and/or their metabolic imprint in the gut and then function at distant sites, including the skin, airways, heart, brain, and metabolism [72]. Inspired by this systemic behavior, the proper use of probiotics in the oral cavity or gut could be a promising application in periodontitis treatment.

### Probiotics’ potential use in periodontitis

Factors such as diet, drug consumption, environment, host genetics, mode of delivery, or phenotype can influence the high microbial diversity [73]. Our results showed that microbiota and metabolites in the oral cavity change when people have periodontitis. In this respect, efforts can be made to explore the use of probiotics to modulate the composition of plaque as monotherapy for the prevention of chronic periodontitis and gingivitis or as adjunctive therapy with scaling and root planing in treatment for chronic periodontitis. Studies showed that probiotics provide beneficial effects for periodontal parameters, including plaque index, gingival index, bleeding on probing, clinical attachment level, gingival crevicular fluid volume, and host response factors [74]. As two known classes of probiotics, lactic acid bacteria (LAB) and bifidobacteria are most frequently and successfully used in treatment of diseases related to the gut microbiota and oropharyngeal infections [75]. However, their role in modulating periodontal diseases is not fully understood. Rostyslav et al. [76] studied specific properties of six LAB strains and two bifidobacteria strains with respect to their resistance to antibiotics, resistance to biological agents (gastric juice, bile, and pancreatic enzymes), and adhesive properties, providing us with a comprehensive approach for assessing properties and selecting the “best” probiotic strains for the treatment of periodontitis.

### PPPM concept in the current study

GCP samples are noninvasively accessible and easily processed. In addition to pathophysiological roles, oral microflora and metabolites in oral samples are valuable biomarkers for patient stratification, disease monitoring, predictive diagnostics, and targeted prevention [77, 78]. Predictive services might then be provided considering several potential relative diagnoses: directly for periodontal diseases and indirectly for disorders of the digestive tract, obesity, immunity diseases, and cancer.

Moreover, multi-omics might be a best choice for advanced diagnostic tools specifically utilizing liquid biopsies since these approaches are well justified by several recent studies [79]. 16s RNA gene sequencing and GC-MS tools assist us in determining how many bacteria species are present in a specimen, the quantity and ratio of each species, and the imputed functions and metabolic “fingerprint” of the gene expressions detected, suggesting that any patient’s individual microbiome and metabolic information can be identified [2].

For individuals, through the multi-omics analysis of GCF, we can determine unique oral bacteria species and metabolite information and then analyze the related molecular pathway in global terms, which can predict whether there is any disorder in periodontal tissue or system health. Specifically in terms of the results of this study, if the most direct biomarkers citramalic acid and N-carbamylglutamate in GCF both increase greatly compared with the previous ratio or are at a high level, the individual could be at risk of periodontitis or already have periodontitis or other related latent diseases. This forecasting then triggers targeted prevention early in life, such as increased attention to oral hygiene, mastering the correct method of brushing teeth, maintaining a healthy diet, adopting a healthier life style, or receiving more regular physical examinations [80]. If any disorder in oral microbiota appears or a disease is diagnosed, it may also help people develop a personalized treatment strategy such as the selection of proper individualized probiotic species. In populations, understanding microbiome activity and characterizing commonly used biomarkers for periodontitis are essential to developing future strategies of public healthcare and potentially providing information for probiotics development.

### Strengths and limitations

Biological omics enable the research and treatment of diseases to shift from a single-parameter model to a multiparameter model [81]. At the same time, the PPPM strategy in oral health requires multi-omics integration analysis to systematically explore the molecular mechanisms and detect effective biomarkers [82].

However, the limitation of this study relates to sample collection from a single region at only one time. A further cohort investigation with samples from patients with different extents of periodontal destruction from a wide geographical area may be required for a better understanding of the dynamic shifts in the microbiome composition and function and the changes in metabolism during disease development.

## Conclusions

In conclusion, dysbiosis in the polymicrobial community structure and changes in metabolism could be mechanisms underlying periodontitis. Our findings provide scientific evidence for an in-depth understanding of the relationship between the oral microbiota and metabolism and identify effective biomarkers in the GCF of periodontitis, pointing to a potentially effective strategy for the diagnosis, prognosis, and management of periodontal therapy.

## Electronic supplementary material

ESM 1 α diversity metrics in samples from patients with GCP (Group P) and healthy controls (Group N), as determined by the Chao1 index, ACE index, Shannon index and Simpson index. (JPG 1313 kb)

ESM 2 (JPG 404 kb)

ESM 3 **(a)** Bar graphs of taxa at different levels according to the relative abundance in samples. **(b)** Relative abundance of the microbial composition at different levels in patients with GCP (Group P) and healthy controls (Group N). (JPG 255 kb)

ESM 4 Functional pathways of metabolism in microbial communities were predicted from an OTU table using the PICRUSt algorithm with references from the KEGG database. Differentially abundant pathways between the GCP group (P) and healthy group (N) based on Student’s t test are shown in colored circles (*P* < 0.05). (JPG 470 kb)

ESM 5 (XLSX 17 kb)

ESM 6 (XLSX 13 kb)
