# Supplementary figures and images for: Microbial and metabolomic analysis of gingival crevicular fluid in general chronic periodontitis patients: lessons for a predictive, preventive, and personalized medical approach
Source: EPMA J. 2020 Apr 16;11(2):197–215. doi: 10.1007/s13167-020-00202-5 (PMC7272536; doi:10.1007/s13167-020-00202-5)

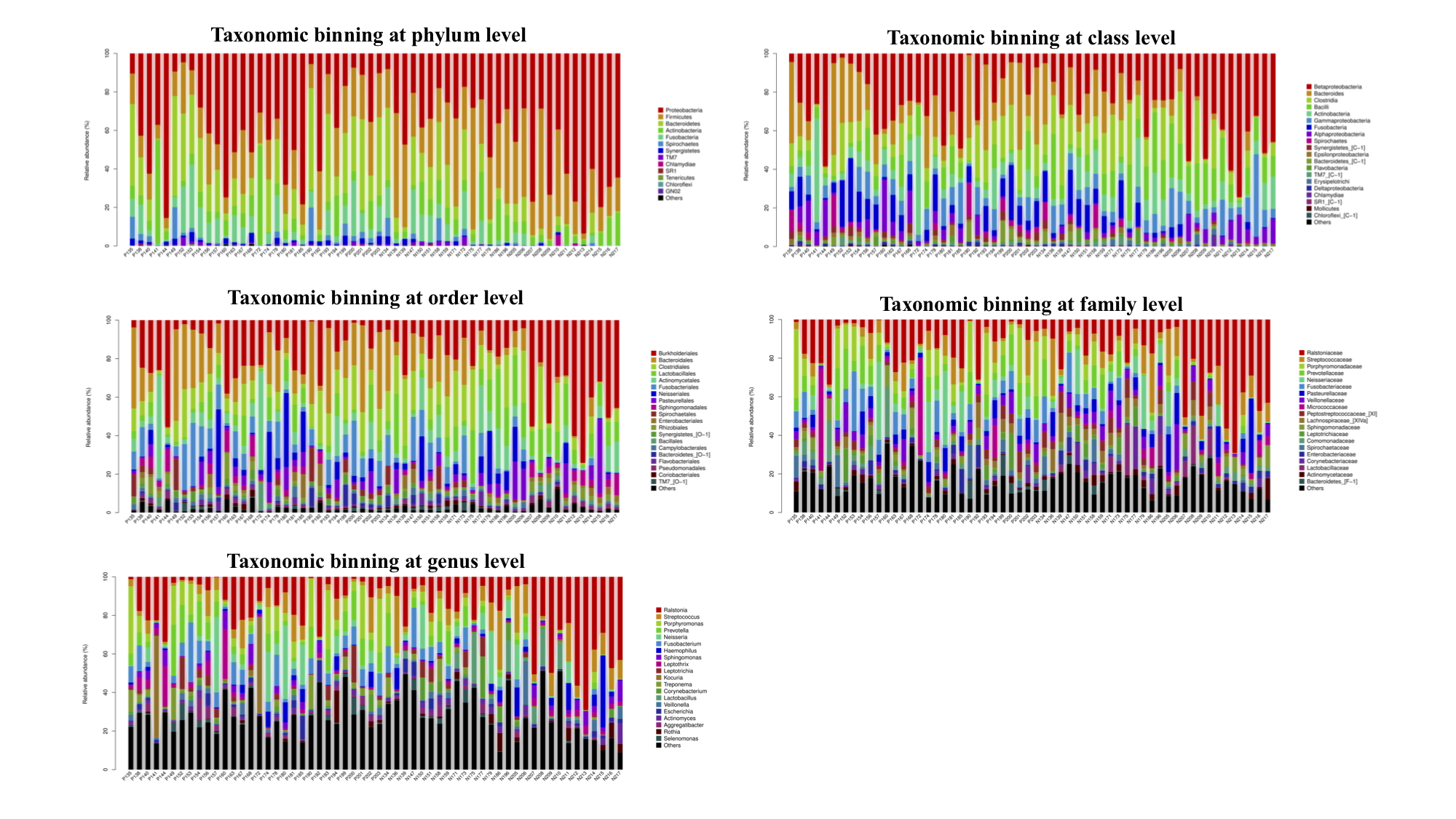

Supplement: Supplementary file 1 — α diversity metrics in samples from patients with GCP (Group P) and healthy controls (Group N), as determined by the Chao1 index, ACE index, Shannon index and Simpson index. (JPG 1313 kb) [file 13167_2020_202_MOESM1_ESM.jpg]

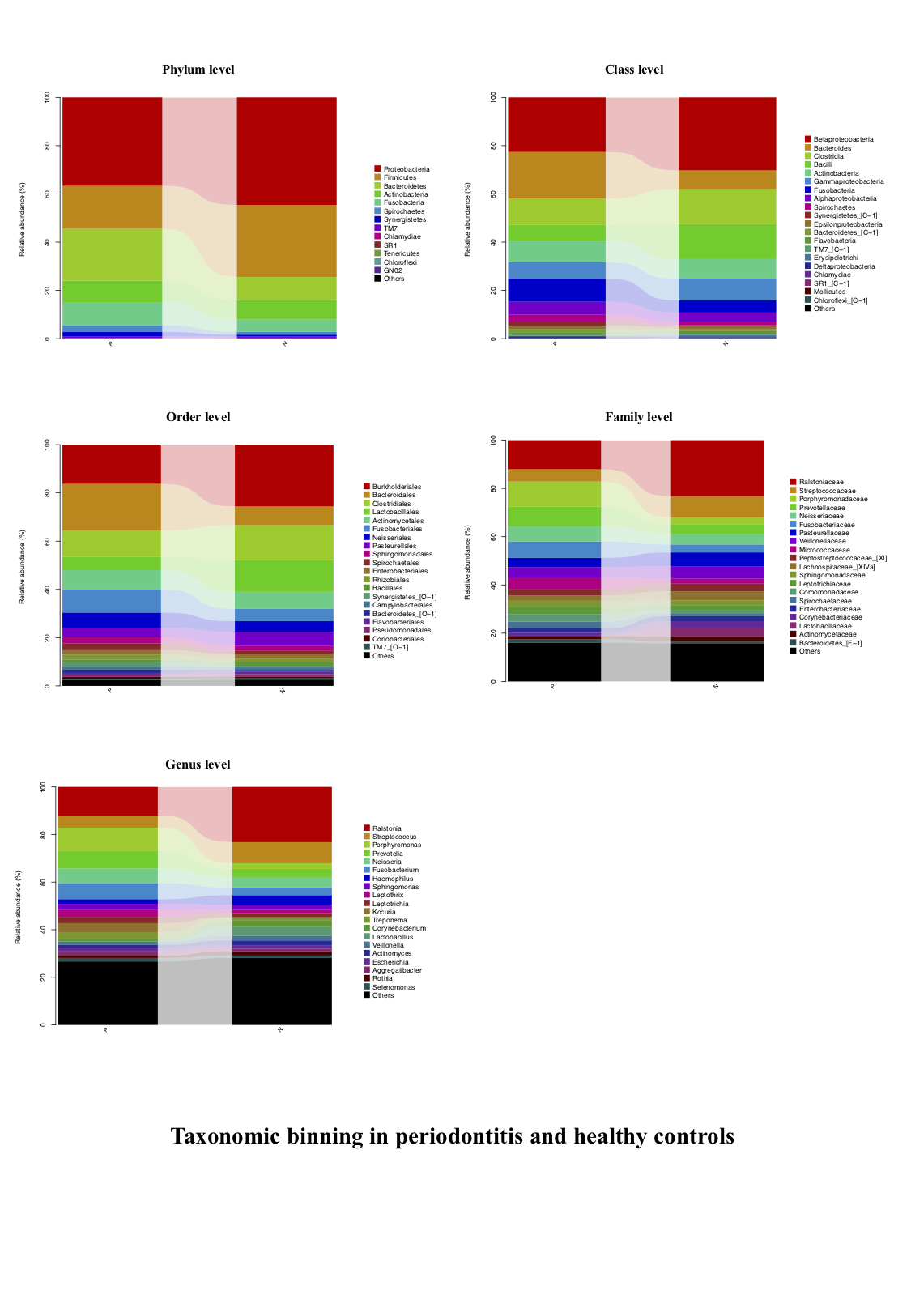

Supplement: Supplementary file 2 — (JPG 404 kb) [file 13167_2020_202_MOESM2_ESM.jpg]

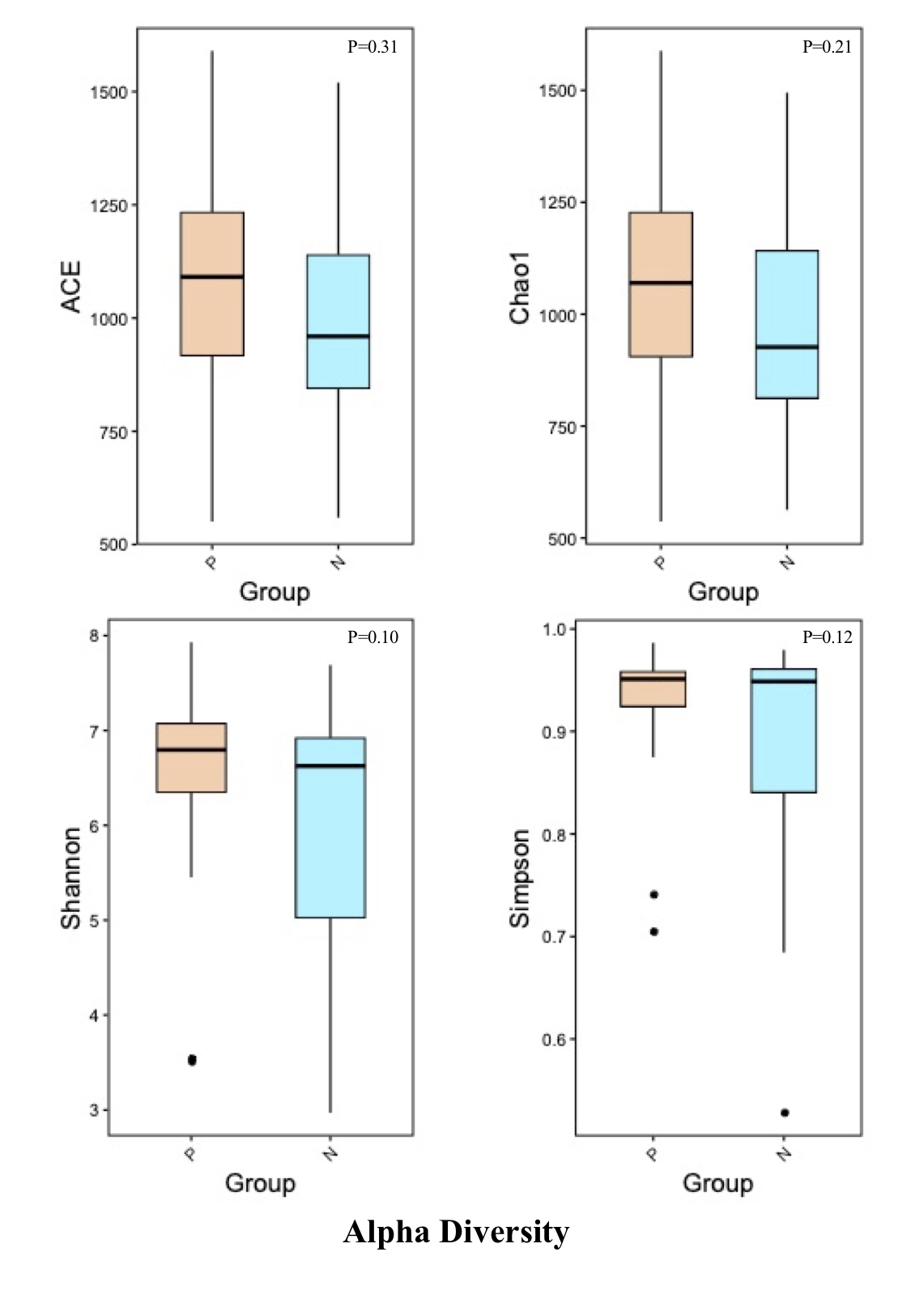

Supplement: Supplementary file 3 — (a) Bar graphs of taxa at different levels according to the relative abundance in samples. (b) Relative abundance of the microbial composition at different levels in patients with GCP (Group P) and healthy controls (Group N). (JPG 255 kb) [file 13167_2020_202_MOESM3_ESM.jpg]

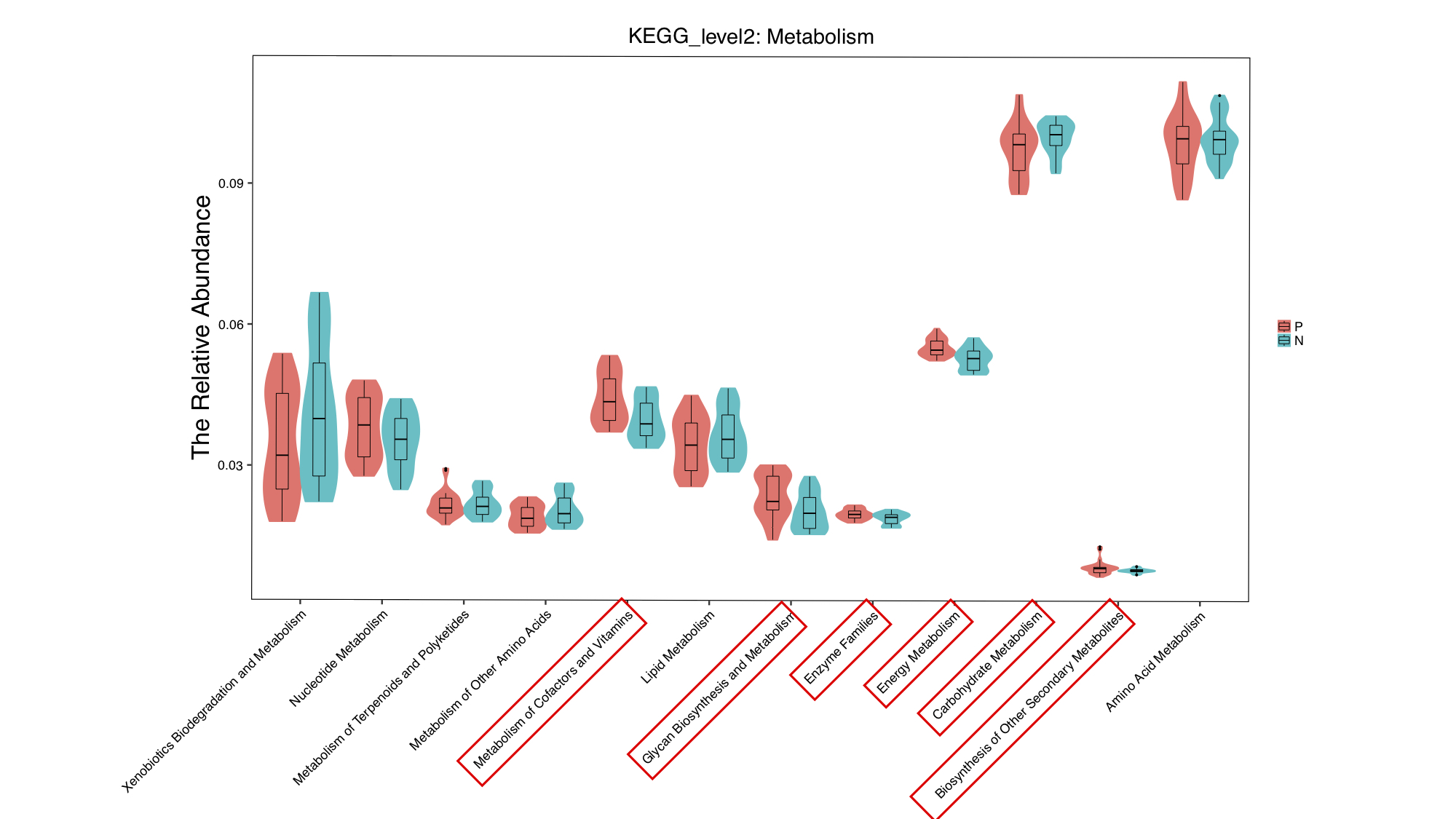

Supplement: Supplementary file 4 — Functional pathways of metabolism in microbial communities were predicted from an OTU table using the PICRUSt algorithm with references from the KEGG database. Differentially abundant pathways between the GCP group (P) and healthy group (N) based on Student’s t test are shown in colored circles (P < 0.05). (JPG 470 kb) [file 13167_2020_202_MOESM4_ESM.jpg]
